# Morphological Analysis of the Latissimus Dorsi Tendon Insertion: Implications for Optimizing Tendon Transfer Surgeries in Rotator Cuff Repair

**DOI:** 10.1002/ca.24285

**Published:** 2025-05-14

**Authors:** Sehyun Kwon, Chang Hyuk Choi, Hongtae Kim, Mi‐Sun Hur

**Affiliations:** ^1^ Daegu Catholic University School of Medicine Daegu Republic of Korea; ^2^ Department of Orthopedic Surgery, Daegu Catholic University Medical Center Daegu Catholic University School of Medicine Daegu Republic of Korea; ^3^ Department of Anatomy Daegu Catholic University School of Medicine Daegu Republic of Korea

**Keywords:** latissimus dorsi, rotator cuff tear, tendon transfer, teres major

## Abstract

Latissimus dorsi (LD) tendon transfer is widely used to treat irreparable rotator cuff tears, particularly in cases with posterosuperior and anterosuperior tear patterns. We conducted a comprehensive anatomical analysis of the attachment of the LD tendon to the humerus, focusing on its morphological patterns and morphometric features, which are critical for optimizing the surgery. Dissection of 32 LD tendons in 16 Korean cadavers revealed three insertion patterns: fully combined (40.6%), partially combined (34.4%), and fully separated (25.0%). The mean width, length, and thickness of the tendons were 48.4, 56.2, and 6.2 mm, respectively. There were significant asymmetries in tendon dimensions. There were also tendinous slips connecting the LD tendon to the triceps tendon, adjacent brachial fascia, coracoid process, and anterior joint capsule of the shoulder in nine specimens, emphasizing the anatomical variability relevant to surgical planning. These findings provide insights for determining surgical approaches on the basis of patient anatomy, and whether to use LD transfer alone or combined LD and teres major transfers. Understanding the anatomical variations will help to make interventions more precise, which in turn should enhance the efficacy of tendon transfer procedures and improve functional outcomes for patients with complex shoulder pathologies.

## 1 Introduction

Rotator cuff tears affect approximately 21% of the general population, their incidence increasing with age (Yamamoto et al. 2010). The choice of surgical or nonsurgical treatment depends on patient age, injury chronicity, and imaging results. Nonsurgical treatments are often sufficient for older patients with low functional demands and irreparable tears. However, surgical interventions typically yield better outcomes for younger patients with high functional demands. These include rotator cuff repair as the primary treatment for repairable tears, and procedures such as latissimus dorsi (LD) transfer for irreparable rotator cuff tears, or in addition to a reverse shoulder arthroplasty to correct a deficit (Guadagno et al. 2023).

Surgical treatments for rotator cuff tears are planned on the basis of their location and severity. Irreparable rotator cuff tears are typically categorized into posterosuperior and anterosuperior patterns. Posterosuperior tears are more common and include complete tears of the supraspinatus, infraspinatus, and teres minor muscles. Anterosuperior tears comprise complete tears of the supraspinatus and subscapularis muscles, sometimes accompanied by injury to the biceps long head (Millett et al. 2008). LD tendon transfer is an effective surgical optimization for patients with irreparable posterosuperior or anterosuperior rotator cuff tears, and often improves shoulder function (Nicholson et al. 2022; Baek et al. 2024; Berthold et al. 2024; Kany et al. 2024). The LD tendon can be anchored at multiple target positions depending on individual patient pathology. The tendon is anchored into the lesser tuberosity for isolated irreparable subscapularis tears, and into the anterior aspect of the greater tuberosity for combined irreparable subscapularis and supraspinatus tears (Elhassan et al. 2020; Lafosse et al. 2020).

Pectoralis major transfer has been the standard procedure for irreparable subscapularis tears and anterior superior rotator cuff tears, but it has limitations in maintaining internal rotation strength and range of motion. In contrast, LD transfer offers biomechanical advantages by addressing vector alignment issues, thereby leading to improved shoulder function. Recent studies have highlighted LD transfer as a promising option with better short‐term outcomes, so it is increasingly favored in complex cases (Mun et al. 2018; Reid et al. 2024).

LD tendon transfer is a versatile surgical technique that can be performed alone or in combination with other procedures such as teres major (TM) transfer and reverse shoulder arthroplasty. The reported effectiveness of these techniques is variable, some studies suggesting that combining LD and TM transfers provides superior strength and others indicating that LD transfer alone provides better functional outcomes. Kany and Selim (2019) found that combining LD and TM transfer provides greater strength than LD transfer alone. The greater bulk of the TM reduces its risk of tendon necrosis or rupture; the LD is relatively thin. In contrast, Lichtenberg et al. (2012) found that combined LD and TM transfers and LD transfers alone both achieved good functional results for massive irreparable posterosuperior rotator cuff tears. LD transfer alone improved abduction and flexion, while the combined transfer only enhanced abduction strength. However, Lichtenberg et al. (2012) found that cuff tear arthropathy progressed only after the combined transfer, suggesting that compromising both anterior stabilizers (LD and TM) during the procedure increases the risk of anterior superior escape, thereby increasing the risk of such arthropathy. These differences in findings suggest that the optimal surgical approach differs among patients and their surgical goals. Some patients could benefit more from LD transfer alone, while in others the outcomes would be better treated by combining LD and TM transfer.

Patel et al. (2022) found that patients undergoing reverse total shoulder arthroplasty with LD transfer showed improved range of motion, functional scores, and pain level at mid‐term follow‐up, and had low rates of shoulder‐related complications. Baek et al. (2022) found that reverse shoulder arthroplasty combined with anterior LD and TM tendon transfer can effectively restore both active elevation and internal rotation in patients with cuff tear arthropathy and irreparable massive rotator cuff tears. These studies emphasized the versatility and effectiveness of LD tendon transfer in addressing complex shoulder pathologies, showing that the procedure provides significant functional benefits and improved patient outcomes. The choice between isolated and combined transfers should be based on the needs and anatomical considerations of the patient.

Understanding the anatomical characteristics of the LD and its relationships with nearby muscles is essential for improving both LD alone and combined LD and TM transfer techniques. The surgical approach can vary according to the patterns of LD tendon attachments to the humerus. Knowledge of the insertion width and the thickness and length of the LD tendon can therefore be important for precise surgical planning. There are diverse descriptions of the anatomy of the LD insertion on the humerus. Standring (2020) stated that the LD attaches to the floor of the intertubercular groove of the humerus and that its attachment extends higher on the humerus than the attachment of the TM. Dancker et al. (2017) consistently found that insertions for the TM and LD tendons were separate, the former attaching directly to the crest of the lesser tubercle and the latter inserting anterior to the TM tendon. In contrast, Cleeman et al. (2003) found that the LD tendon typically overlapped the superior 39% of the TM tendon insertion. These variations emphasize the importance of recognizing individual anatomical differences to achieve optimal surgical outcomes.

In this study, we performed a comprehensive analysis of the LD tendon and its morphological patterns of attachment to the humerus, along with its morphometric features. The aim of this investigation of anatomical characteristics is to improve surgical techniques for LD alone and combined LD and TM transfers, optimize patient outcomes, and offer insights into the most effective approaches for addressing various rotator cuff pathologies. Understanding the anatomical variations will facilitate more customized and precise surgical interventions, ultimately enhancing the efficacy and reliability of strategies for rotator cuff repair.

## 2 Materials and Methods

**FIGURE 1:**
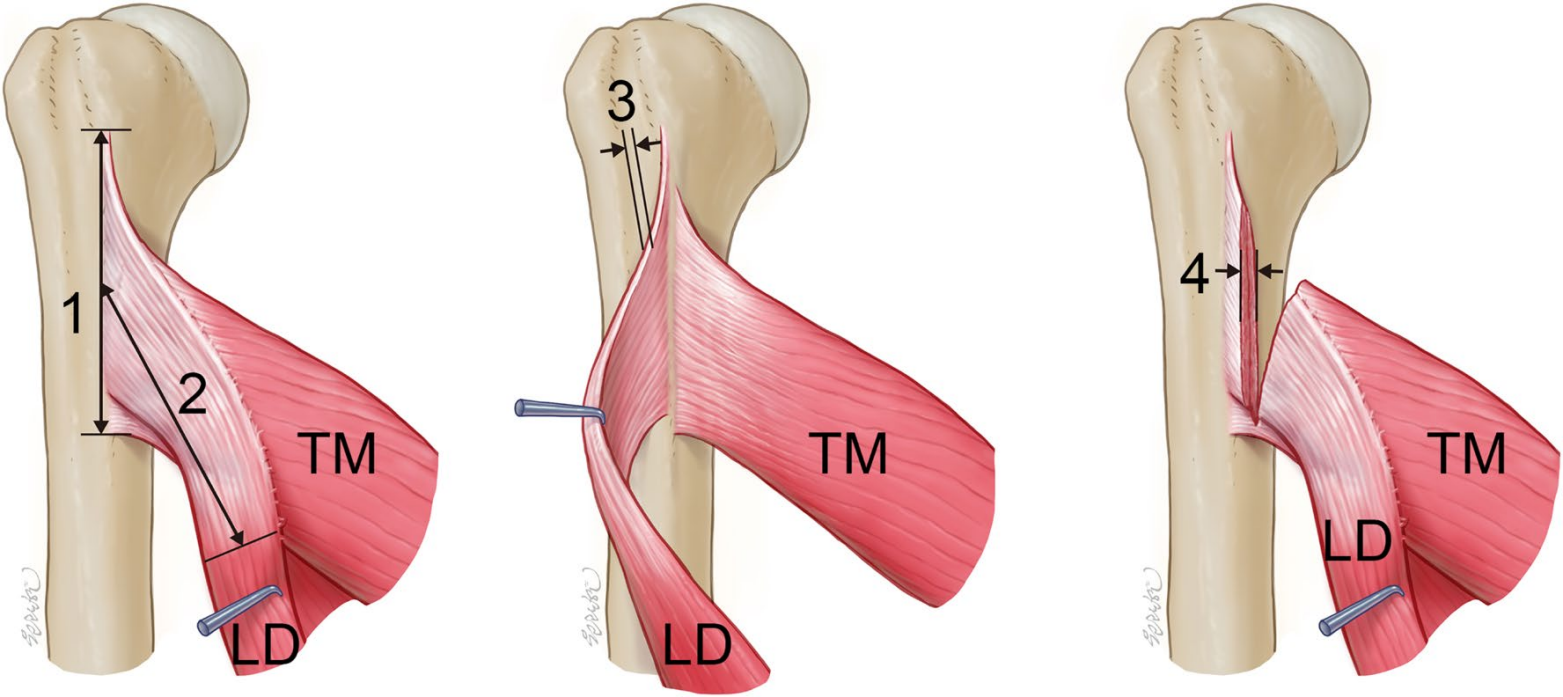
Measurements of the latissimus dorsi (LD) tendon at its humeral attachment. 1. Width of the LD tendon at its insertion on the humerus, 2. Length of the LD tendon, 3. Thickness of the LD tendon in fully separated LD and teres major (TM) tendon patterns, 4. Thickness of the LD tendon in fully combined or partially combined patterns.

Thirty‐two LD muscles in 16 embalmed adult Korean cadavers were examined (11 males, five females, mean age at death 77.7 years, range 61–92 years). The pectoralis major and deltoid muscles were carefully dissected so they could be examined in more detail. The dissection focused primarily on the insertions between the LD and TM tendons and their humeral attachments. For the morphometric analysis, a Digital Electronic Caliper (Fine Science Tools, Heidelberg, Germany) was used to measure the mean width, length, and thickness of the LD tendon at its point of attachment to the humerus (Figure 1). The humerus was measured with a tape measure from the inferior point of the tuberosity to the medial epicondyle to examine the relationship between muscle dimension and cadaver height. Various tendinous slips were identified and recorded.

All cadavers had been legally donated to Daegu Catholic University School of Medicine. This study was conducted in accordance with the Declaration of Helsinki. No transplant donors were from a vulnerable population and all donors or their next of kin voluntarily provided written informed consent. The study was approved by the Institutional Review Board of the Daegu Catholic University (no. CR‐23‐022).

## 3 Results

**FIGURE 2:**
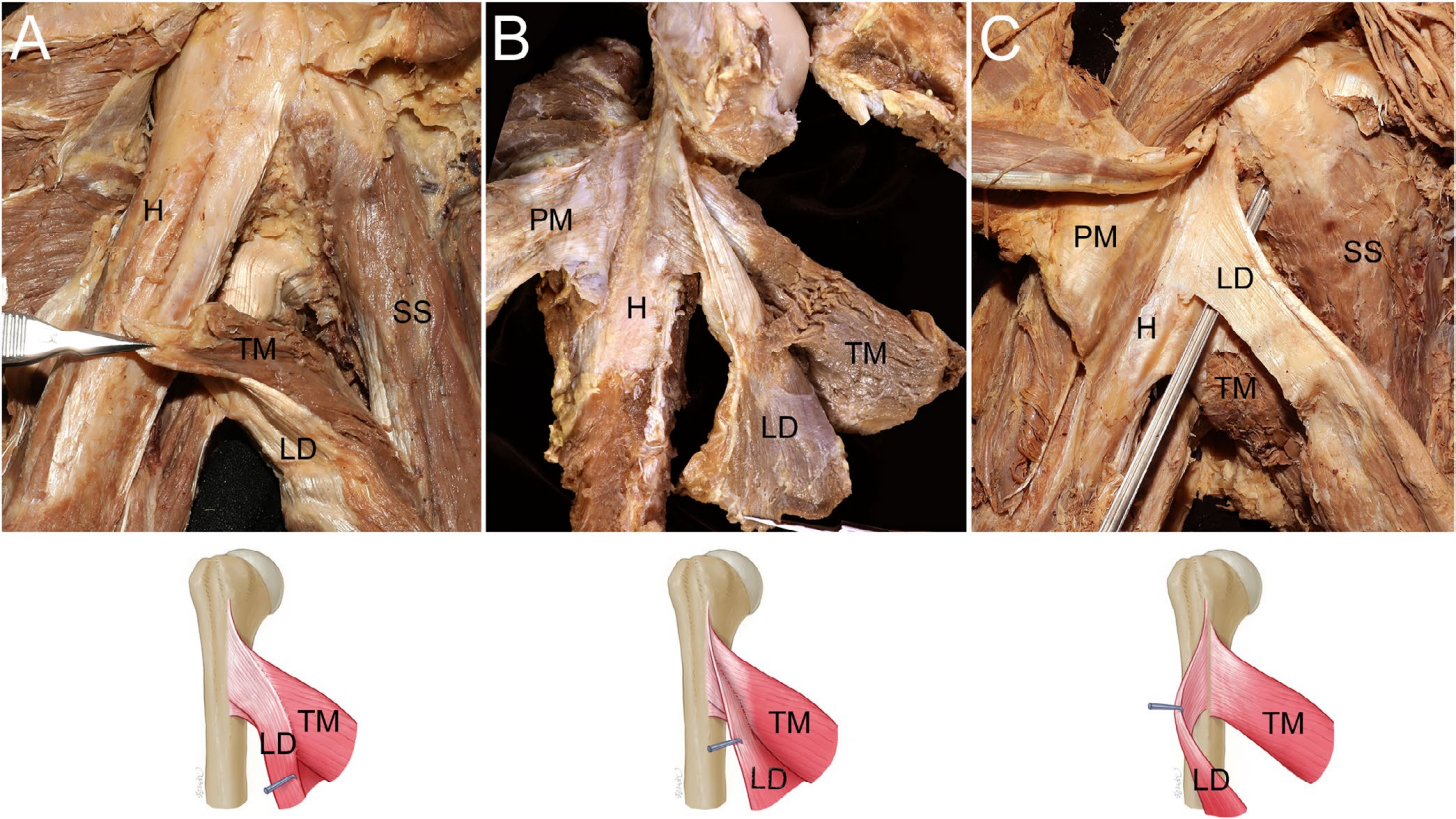
Insertion patterns of the LD and TM based on their combined configurations. (A) Fully combined pattern: The LD tendon and TM fibers are completely fused before inserting into the humerus (H), becoming fully integrated as they approach the insertion point. The combined LD tendon and TM fibers were severed at the insertion site and pulled anteroinferiorly to expose their components. (B) Partially combined pattern: The LD tendon and TM fibers are partially fused obliquely and do not merge fully before their insertion into H. The insertion areas of the LD and TM were cut, the LD tendon being reflected anteriorly to display their partial fusion. (C) Fully separated pattern: The LD tendon and TM fibers insert separately into H, as indicated by the instrument. PM, pectoralis major; SS, subscapularis.

The LD fibers formed a flat tendon by inserting into the intertubercular groove of the humerus, while the TM fibers attached directly to the groove via tendon and muscle fibers. The insertion patterns of the LD in conjunction with the TM were categorized into three types on the basis of their combined morphologies. Fully combined, partially combined, and fully separated patterns were observed in 13 (40.6%), 11 (34.4%), and eight (25.0%) of the 32 specimens, respectively (Figure 2). The LD and TM tendons were often connected at the lower edge in the partially combined pattern.

**FIGURE 3:**
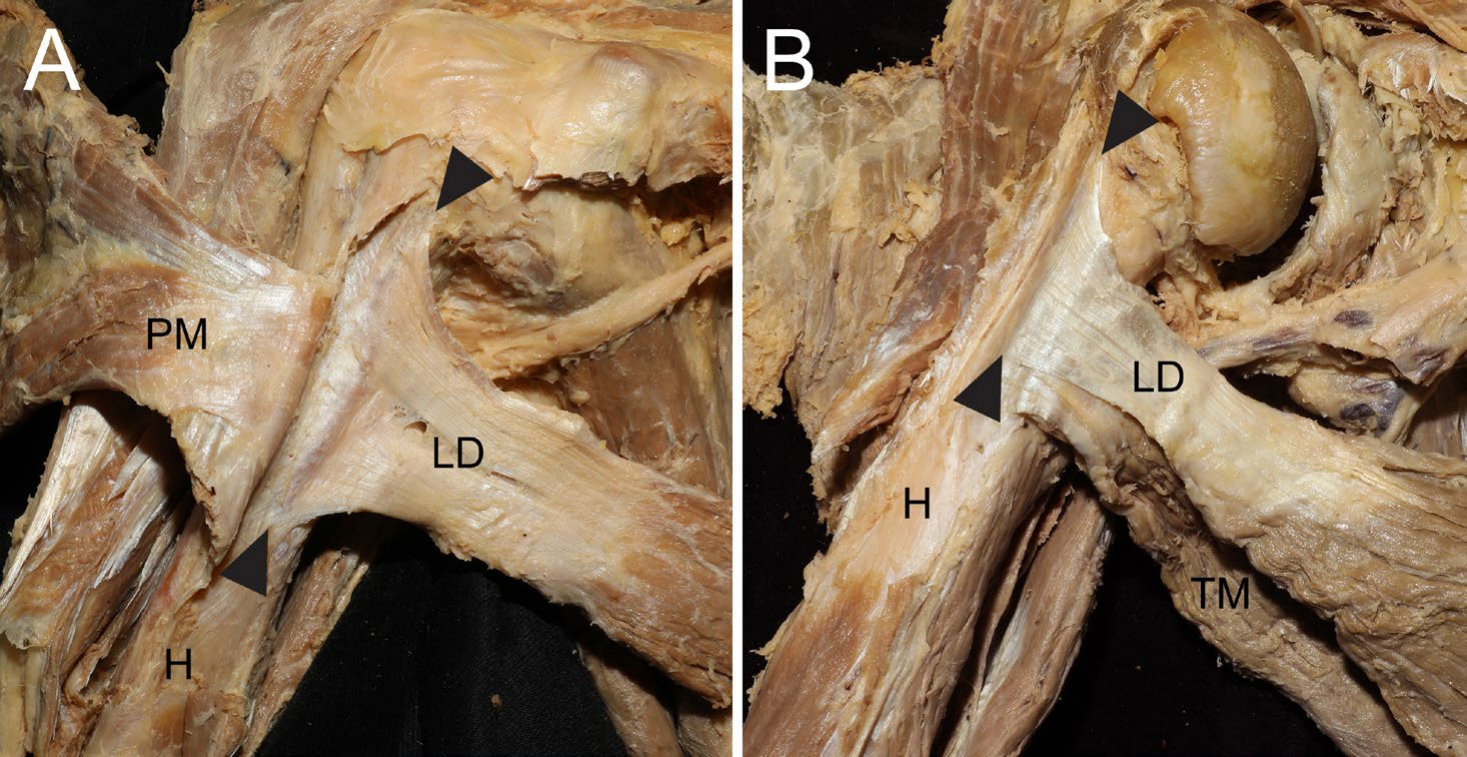
The maximum and mean widths of the LD tendon insertion into H. (A) The maximum insertion width of the LD tendon was 67.3 mm. (B) The mean insertion width of the LD tendon was 43.5 mm. The insertion widths of the LD tendon at H were measured at the locations indicated by the arrowheads.

The dimensions of the LD tendon at its humeral attachment point were: width, 48.4 ± 11.7 mm (mean ± SD) (range: 28.0–67.3 mm; left: 47.5 ± 11.7 mm, right: 49.4 ± 11.9 mm); length, 56.2 ± 11.3 mm (range: 34.8–84.0 mm; left: 58.2 ± 10.2 mm; right: 54.2 ± 12.3 mm); thickness, 6.2 ± 5.2 mm (range: 1.0–25.1 mm; left: 6.6 ± 6.4 mm; right: 5.7 ± 3.7 mm) (Figure 3). The left and right LD tendons differed in width by > 10 mm in three male cadavers, and there were asymmetries of 5–10 mm in five male and one female cadaver. There was a length asymmetry of > 20 mm in one male cadaver, and one of > 10 mm in three male and two female cadavers. A thickness asymmetry of > 10 mm was found in two male cadavers; in one, it exceeded 20 mm. The fully separated pattern of the LD tendon had a thickness and width of 1.6 ± 0.6 mm and 40.3 ± 7.4 mm, respectively. In contrast, both the partially and fully combined patterns had mean thickness and weight of 7.7 ± 5.1 mm and 51.2 ± 11.6 mm, respectively. The humeral length was 25.1 ± 1.5 mm (range: 21.7–27.4 mm; left: 25.3 ± 1.4 mm; right: 25.0 ± 1.6 mm) (Table 1).

**TABLE 1:** Characteristics and asymmetry of the latissimus dorsi (LD) tendon at the humeral attachment and the mean humeral length (unit: mm).

Characteristic	Overall	Left	Right	Notes
LD tendon width	48.4 ± 11.7 (28.0–67.3)	47.5 ± 11.7	49.4 ± 11.9	Asymmetry > 10 mm in three males Asymmetry > 5 and ≤ 10 mm in six cadavers (five males and one female)
LD tendon length	56.2 ± 11.3 (34.8–84.0)	58.2 ± 10.2	54.2 ± 12.3	Asymmetry > 20 mm in one male Asymmetry > 10 mm in five cadavers (three males and two females)
LD tendon thickness	6.2 ± 5.2 (1.0–25.1)	6.6 ± 6.4	5.7 ± 3.7	Asymmetry > 20 mm in one male Asymmetry > 10 mm in one male
LD tendon thickness (separated pattern)	1.6 ± 0.6	—	—	—
LD tendon width (separated pattern)	40.3 ± 7.4	—	—	
LD tendon thickness (combined pattern)	7.7 ± 5.1	—	—	Includes partial and fully combined types
LD tendon width (combined pattern)	51.2 ± 11.6			
Humeral length	25.1 ± 1.5 (21.7–27.4)	25.3 ± 1.4	25.0 ± 1.6	Measured from the inferior point of the lesser tubercle to the medial epicondyle of the humerus

*Note*: Data are mean ± SD or mean ± SD (range) values in millimeters.

**FIGURE 4:**
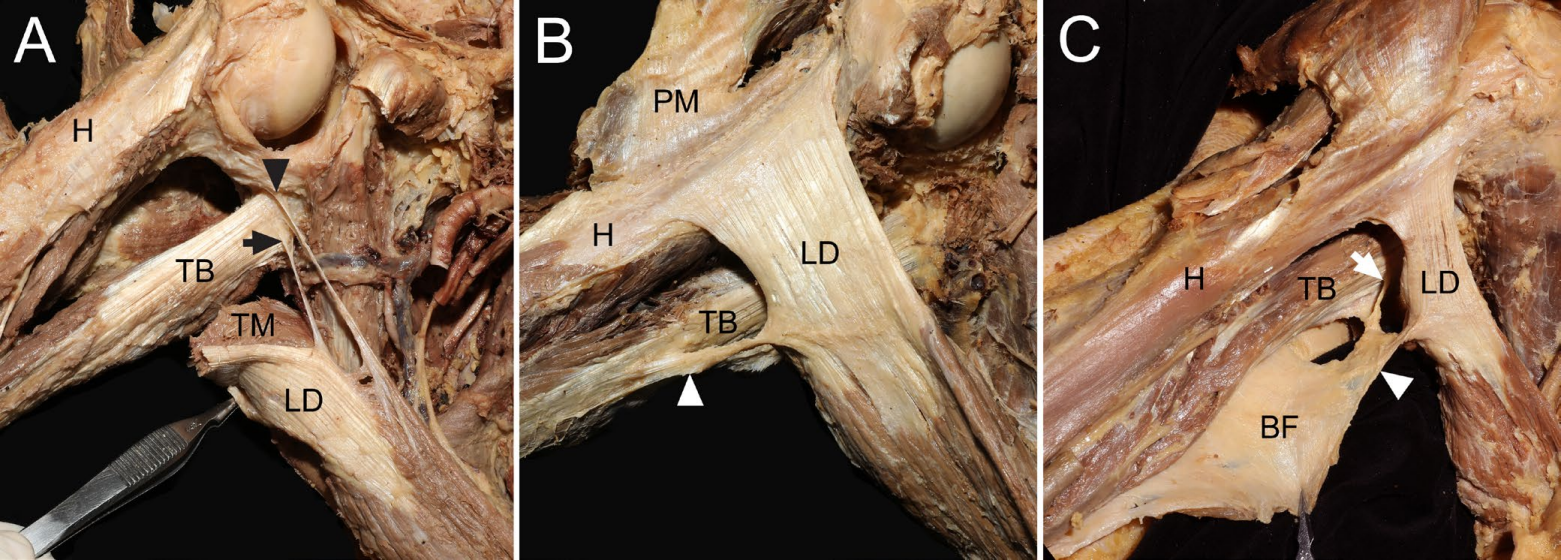
Various tendinous slips of the LD. (A) The tendinous slips of the LD tendon inserted into the anterior joint capsule of the shoulder (arrowhead) and the triceps brachii (TB) tendon (arrow). The combined LD and TM tendon fibers were cut at their insertion sites and drawn anteroinferiorly to reveal the tendinous slips. (B) Tendinous slip (arrowhead) serving as a link between the LD and TB tendons. (C) Tendinous slips of the LD tendon inserted into the brachial fascia (BF, arrowhead) and TB tendon (arrow).

Tendinous slips of the latissimus dorsi (LD) were found in nine of the 32 tendons (28.1%) (Figure 4). These slips, which originated from the LD tendon or its adjacent muscle fibers, connected to the triceps tendon, the surrounding brachial fascia, and the coracoid process. Notably, one specimen had two slips from the LD tendon and the adjacent LD fibers that attached to the triceps tendon and anterior joint capsule of the shoulder. A unilateral tendinous slip was found in seven cadavers, and there was a bilateral slip in one.

## 4 Discussion

The two currently‐used types of LD transfer procedure are the L'Episcopo procedure, which combines LD and TM transfer, and a modified version that only uses the LD (Kazum et al. 2022; Valenti et al. 2022). Our anatomical investigation showed that fully and partially combined tendon patterns comprise approximately 75% of cases, indicating that the standard L'Episcopo procedure is more often applicable for tendon transfer.

Their overlapping positions can make the LD and TM tendons difficult to distinguish using CT or MRI, posing a challenge for preoperative planning. The frequencies of the three insertion patterns (fully combined 40.6%, partially combined 34.4%, and fully separated 25.0%) showed no significant difference, none being overwhelmingly dominant. This relatively small variation suggests that all insertion types should be considered in planning surgical strategies. To optimize LD and combined LD‐TM transfers in irreparable rotator cuff tears, surgeons should design fixation strategies on the basis of insertion type. A single fixation site could suffice in fully combined insertions, while partially combined or fully separated tendons often require independent fixation points to preserve biomechanical integrity. Given the variability of tendon dimensions and morphology, intraoperative assessment is essential for achieving appropriate tensioning and anatomical restoration. When the tendons are separated, individual repositioning allows for greater precision in alignment and fixation, while combined tendons require unified handling, which limits intraoperative flexibility.

We found mean tendon thicknesses of 1.6 and 7.7 mm in the separated and combined patterns, respectively, a nearly fivefold difference between the insertion types. This variation significantly influences the tendon transfer surgery approach, as the morphometric differences affect both the mechanical strength and flexibility achieved by the transfer. Integrated tendons could require the surgeon to adjust fixation methods or adapt the positioning of the transferred tendon when their pattern is fully or partially combined, as when isolated subscapularis tears or anterior superior rotator cuff tears are addressed. It is important to clarify whether the measured thickness in the combined and partially combined patterns reflects the contributions of both the LD and TM tendons. In the completely separated pattern, the mean thickness of the LD tendon was only 1.6 mm, whereas in the combined and partially combined patterns it was 7.7 mm. This substantial difference suggests that the TM tendon contributes significantly to the overall thickness—and probably to the mechanical strength—of the combined unit. Recognizing this contribution is essential for surgical planning and optimizing fixation strategies.

Our findings both align and contrast with previous research. Goldberg et al. (2009) also found three insertion patterns: completely separate (eight of 12 cadavers, 67%), loosely bound (three cadavers, 25%), and completely joined (one cadaver, 8%). However, we found these patterns in the opposite order of prevalence: fully separated (25.0%), partially combined (34.4%), and fully combined (40.6%). Goldberg et al. (2009) also found the mean width of the LD tendon at its insertion point to be 3.3 cm and its mean length 7.3 cm. Gates et al. (2020) found a width of 29.3 mm at the humeral insertion, while Cleeman et al. (2003) found a mean width of 4.2 cm. Comparatively, the tendons in our study were wider and shorter, with a mean width of 48.4 mm and a mean length of 56.2 mm (Table 2). These discrepancies could be attributable to differences in sample size, race, and preservation method among the study populations.

**TABLE 2:** Comparison of anatomical patterns and measurements across studies.

Study	Completely separate	Partially combined	Fully combined	Mean width	Mean length
Goldberg et al. (2009)	66.7% (8 of 12 cadavers)	25.0% (3 cadavers)	8.3% (1 cadaver)	3.3 cm	7.3 cm
Gates et al. (2020)	N/A	N/A	N/A	2.93 cm	N/A
Cleeman et al. (2003)	N/A	N/A	N/A	4.2 cm	N/A
Present study	25.00%	34.40%	40.60%	4.84 cm	5.62 cm

The insertion patterns of the LD and TM tendons in our findings could help inform the choice between an arthroscopic and an open approach for tendon transfer surgery. In cases with a fully separated LD and TM tendon pattern (25.0%), an arthroscopic approach could be more feasible, as the separation allows for individual tendon handling and potentially more precise adjustment within the limited visual field of minimally invasive procedures. In contrast, when the tendons are fully combined (40.6%) or partially combined (34.4%), an open approach could offer advantages, as these configurations present a single, integrated tendon unit that could be more easily managed and positioned under direct visualization.

Regarding surgical risks, Kany and Selim (2019) emphasized the potential for nerve injuries, including to the radial and/or axillary nerves and also the circumflex vessels. They noted that using whip stitching for combined LD and TM tendon transfers is the most challenging aspect of shoulder arthroscopy. Gates et al. (2020) examined the positions of the axillary and radial nerves relative to the LD tendon in various shoulder positions in cadavers with the aim of minimizing iatrogenic nerve injury risk by informing safer surgical techniques. Goldberg et al. (2009) similarly found that the axillary and radial nerves occupied consistent positions near the LD tendon insertion in all specimens. They specifically noted that the axillary nerve was slightly posterior and proximal to the LD insertion and covered by a fibrous sheath, suggesting that flexing the shoulder and internally rotating the arm will improve the safety of the surgical approach. The radial nerve was located anterior and distal to the LD tendon insertion and covered by fat. Cleeman et al. (2003) also investigated the relationship between these nerves and various arm positions to provide further guidance for avoiding nerve injury during surgery.

Tendinous slips of the LD were found in 28.1% of the specimens in the present study. These slips connected to the triceps tendon, adjacent brachial fascia, and anterior joint capsule of the shoulder. These anatomical variations have clinical significance since they could influence the approach to and outcomes of surgical procedures such as tendon transfers. Variations of the axillary arch muscle have been well documented and include tendinous or muscular slips from the LD. The axillary arch, also known as Langer's arch, is typically 7–10 cm long and 5–15 mm wide and is found in 7%–8% of the population. These slips connect to structures such as the triceps brachii long head, pectoralis major, pectoralis minor, coracobrachialis, fascia over the biceps brachii, or coracoid process (Dharap 1994; Loukas et al. 2009; Al Maksoud et al. 2015; Tubbs et al. 2016; Standring 2020). Understanding these variations is essential for optimizing surgical outcomes, minimizing complications, and enhancing the precision of procedures such as tendon transfers and axillary dissections.

The tendinous slips identified in this study could be clinically relevant, particularly in tendon transfer procedures. They could function as secondary attachments that restrict the free excursion of the LD or TM tendons, thereby complicating their mobilization and optimal placement onto the greater tuberosity. Limited tendon excursion is a recognized intraoperative challenge that can adversely affect the functional outcome of tendon transfers. Resection of these tendinous slips could therefore constitute a simple and practical surgical strategy for enhancing tendon mobility and improving excursion. However, further investigation is needed to assess the clinical efficacy and safety of tendinous slip resection as part of tendon transfer procedures.

While combined LD and TM tendon transfer offers enhanced strength, the anatomical variability and specific patterns of the TM tendon remain unclear. A clearer understanding of its morphometry—including insertion dimensions and relationship to the LD tendon—is essential for refining surgical strategies and identifying when TM inclusion is truly beneficial. Although this study highlights the potential advantages of including the TM tendon, no direct morphometric analysis of the TM was performed. Future anatomical research should therefore focus on the TM tendon to establish patient‐specific indications and determine when combined transfer offers the greater functional benefit with minimal complication risk.

This study was conducted using formalin‐fixed cadavers; therefore, an influence of the preservation method on the results cannot be entirely ruled out. The physical properties of formalin‐fixed tissues differ from those of fresh cadavers or living tissue observed during surgery, which can affect the appearance of tendon separation, tissue pliability, and morphometric measurements. Accordingly, differences between our findings and those from fresh cadaver studies or intraoperative observations should be interpreted with caution.

This study was a comprehensive analysis of the morphological patterns and metrics of the LD tendon. It revealed variations that are important for optimizing tendon transfer surgery. The identified patterns of LD and TM tendon insertions and the detailed morphometric data offer valuable insights for preoperative planning and surgical decision‐making. Understanding these anatomical variations will allow surgeons to design more effective procedures that enhance surgical precision and improve outcomes for patients with irreparable rotator cuff tears. Overall, these findings will help to refine current techniques and guide safer and more effective transfers of the LD alone and the LD and TM tendon combined.

## Data Availability

Deidentified data will be made available on reasonable written request submitted to the corresponding author.
